# Cortisol-to-cortisone ratio postpartum is associated with anti-Müllerian hormone a decade later: evidence from a prospective study

**DOI:** 10.1530/EC-25-0871

**Published:** 2026-03-25

**Authors:** Gary Joseph, Elena Colicino, Georgia Dolios, Martha María Téllez-Rojo, Brismar Pinto-Pacheco, Mara Yella, Katherine Svensson, Robert O Wright, Megan Horton, Maria José Rosa, Luisa Torres-Sánchez, Lauren M Petrick

**Affiliations:** ^1^ Department of Environmental Medicine, Icahn School of Medicine at Mount Sinai, New York, New York, USA; ^2^ Center for Nutrition and Health Research, National Institute of Public Health, Cuernavaca, Morelos, Mexico; ^3^ Institute for Exposomic Research, Icahn School of Medicine at Mount Sinai, New York, New York, USA; ^4^ Center for Population Health Research, National Institute of Public Health (INSP), Cuernavaca, Morelos, Mexico; ^5^ The Bert Strassburger Metabolic Center, Sheba Medical Center, Tel-Hashomer, Israel

**Keywords:** anti-Müllerian hormone, cortisol, cortisone, stress, ovarian reserve

## Abstract

**Objective:**

To assess the associations between serum stress-related hormones (cortisol and cortisone) at one month postpartum and AMH levels at 4- and 10-year follow-ups.

**Methods:**

In a subset of 107 postpartum mothers enrolled in the PROGRESS cohort (at a mean age of 29.5 years), we measured serum cortisol and cortisone at one month postpartum via untargeted metabolomics. At 4- and 10-year follow-ups, we measured serum AMH levels. Serum cortisol, cortisone, and the cortisol-to-cortisone (F/E) ratio were analyzed both as continuous variables and as tertiles. The association between serum stress hormones and log_2_-transformed AMH levels was assessed using multivariable linear regression.

**Results:**

The serum cortisol and F/E ratio showed a weak and inverse association with AMH concentration at the 4-year follow-up. However, at the 10-year follow-up, each unit increase in the F/E ratio was associated with a 45% reduction in AMH levels (*β* = −0.60, 95% CI: −1.13; −0.07). In addition, when repeated time points were considered in the model, each unit increase in the F/E ratio was associated with a 32% reduction in AMH levels (*β* = −0.38, 95% CI: −0.75; −0.01). The analysis by tertiles of serum cortisone showed a dose–response relationship with AMH levels at the 10-year follow-up, while the F/E ratio was only associated in the highest tertile at the 4- and 10-year follow-ups, including when considering the model with repeated time points.

**Conclusion:**

Our findings suggest that a higher F/E ratio is associated with a lower AMH level, especially in mothers in the highest tertile of serum stress hormone levels. Our results support the role of stress hormone imbalance in ovarian aging.

## Introduction

Ovarian aging, a central component of the complex biological process of reproductive aging, occurs progressively over time and ultimately leads to menopause – the permanent cessation of menstrual cycles (1). One of the key markers of this process is anti-Müllerian hormone (AMH), a glycoprotein produced by granulosa cells in preantral and small antral follicles (2, 3). AMH levels are widely recognized as a reliable indicator of functional ovarian reserve, reflecting both the quantity and quality of a woman’s remaining oocytes (4). In women, AMH levels increase until approximately 25 years old (5, 6) and then AMH levels gradually decline as women age, signaling a diminishing ovarian reserve and the transition toward menopause (7). This decline serves not only as a marker of aging but also as a predictor of fertility potential and reproductive lifespan (8, 9, 10).

Several factors are thought to influence ovarian aging, such as genetic factors (11), smoking and obesity (12, 13), ovarian surgery (14), and environmental factors (15, 16, 17). Chronic stress exposure can also accelerate the overall body’s natural aging process (18), including ovarian aging (19, 20, 21, 22, 23). In cross-sectional analyses, women with higher serum cortisol levels had significantly lower AMH levels (22); meanwhile, higher urine cortisol levels have been associated with abnormal AMH levels (24) – defined as the values that fell above or below the normative age-specific ranges (24).

Serum cortisol (F)-to-cortisone (E) ratio (F/E ratio) (25) is a marker of cortisol metabolism, mainly of net 11β-HSD activity. 11β-HSD2 converts cortisol to cortisone, which binds mineralocorticoid receptor with lower affinity (26). Evidence on the association between the F/E ratio and AMH levels remains limited. However, it is recognized that elevated intrafollicular F/E ratios are linked to better embryo implantation outcomes in fully unstimulated *in vitro* fertilization (IVF) cycles (25) and to clinical pregnancy among women undergoing IVF treatment. The authors suggest that glucocorticoids may play a role in follicular development and oocyte maturation (27), IVF outcomes, and ovarian health (26).

There are bidirectional and context-dependent effects of cortisol-glucocorticoid receptor signaling on granulosa cell function, including its impact on estrogen production and steroidogenic enzyme expression. Elevated cortisol may accelerate ovarian aging through mechanisms mediated by the glucocorticoid receptor (GR), with effects that vary according to timing, dose, and tissue context. The tissue-specific effects of GRs are determined by the presence of isoforms of GR, GR-α, and GR-β. GR-β does not bind GRs and has inherent transcriptional activity; moreover, it acts as a dominant-negative inhibitor of the transcriptional activity of GR-α (28). Chronic stress and sustained elevation in cortisol can directly disrupt the hypothalamic–pituitary–gonadal axis, leading to decreased secretion of gonadotropin-releasing hormone (GnRH), luteinizing hormone (LH), and follicle-stimulating hormone, which negatively affects follicle development and hastens the depletion of ovarian follicles (29, 30). GRs can indirectly modulate GnRH secretion by alteration of the expression and activation of neuropeptides KISS1 and GnIH, which have opposing effects on GnRH release. High levels of GRs suppress the stimulatory effect of KISS1 while enhancing the GnIH neuron’s function, ultimately inhibiting LH release from the pituitary (28). At the ovarian level, stress-induced levels of GRs can produce ovarian vasoconstriction and granulosa cell apoptosis (by activation of the Fas system) and increase progesterone production in preovulatory granulosa cells; meanwhile, a decrease in LH-induced StAR protein levels in granulosa cells and progesterone production have also been observed (28, 30). Moreover, GRs increase overactivation of primordial follicles (by activation of PI3K/AKT and mTOR pathways, as well as inhibition of SIRT1 pathways), reduce oocytes quality and ovarian reserve by decreasing mRNA expression of GR-gen NR3C1, and impair mitochondrial function and increase reactive oxidative stress (28). Finally, the local concentration and bioavailability of cortisol within the ovarian tissue are modulated by 11β-hydroxysteroid dehydrogenase (11β-HSD) enzymes, and disturbances in their function may lead to higher intrafollicular cortisol (31) and increased insulin resistance (31), which in turn may impair the granulosa cells’ function (31), potentially contributing to a reduction in circulating levels of AMH (32).

Given the limited research on the long-term effects of early postpartum stress, particularly the influence of serum cortisol, cortisone, and the F/E ratio on ovarian aging, there is a critical need to understand how exposure to these hormones during this sensitive window impact AMH levels over time. The Programming Research in Obesity, Growth, Environment, and Social Stressors (PROGRESS), a pregnancy Mexican cohort with a long follow-up of mothers and children, provides a unique opportunity to investigate this relationship a decade after childbirth. The results from this study will contribute to understanding the long-term impact of postpartum stress on ovarian aging and emphasize the early identification of stress-related reproductive risk particularly among women with unexplained infertility or early signs of reproductive aging.

## Methods

This study included a subset of 107 mothers from the PROGRESS cohort with available untargeted metabolomics data on serum cortisol and cortisone one month postpartum and AMH concentration at 4 (*n* = 107) and 10 (*n* = 85) years of follow-up, who were selected to represent the entire age range of mothers in the PROGRESS cohort. The PROGRESS cohort is a prospective study that enrolled 948 pregnant women who received prenatal care at the primary care clinics of the Social Security Institute in Mexico City between July 2007 and February 2011 (33). Women were eligible to participate in the study if they were less than 20 weeks pregnant with a singleton pregnancy, were 18 years old or older, were intended to live in Mexico City for the next three years, had telephone access, did not have a medical history of heart or kidney disease, and were not taking steroids or anti-epileptic medications. They could also not consume alcohol daily or have a drug addiction. At baseline, a structured questionnaire was used for obtaining sociodemographic information: maternal age, maternal level of education, maternal exposure to second-hand smoking at home, and parity. Maternal follow-up is ongoing and began one month after the delivery and continues every two years (34). In each follow-up visit, data on maternal age and maternal anthropometric measurements were also collected. Voluntary written informed consent in Spanish was obtained from each woman before participating in the study.

### Serum stress hormone measurements

Both cortisol and cortisone were measured in serum samples as part of an untargeted analysis with liquid chromatography–high resolution mass spectrometry (LC-HRMS) (35, 36, 37). Serum samples stored at −80°C were thawed on ice, vortexed, and then 35μL aliquots were mixed with 150 μL of ice-cold methanol containing internal standards. Following incubation at −80°C for 30 min to precipitate proteins, the samples were centrifuged and the supernatant was aliquoted and evaporated to dryness. A pooled quality control (‘pooled QC’) sample was generated by combining an additional 75 μL aliquot from 65 randomly selected samples across all analytical batches and then re-aliquoted into 50 μL samples. Following the same protocol, the matrix blank (replacing the plasma with water) and multiple pooled QC samples were extracted and dried. Immediately before LC-HRMS analysis, dried extracts were reconstituted in 100% methanol. Samples were analyzed using reverse-phase (RP) HPLC connected to HRMS in negative mode ionization, as described elsewhere (38). Samples were analyzed in a randomized order with pooled QCs injected routinely throughout the run. Cortisol and cortisone were identified considering retention time, accurate mass, and isotope distribution with reference standards analyzed under the same conditions (retention time <10 s and ppm <20).

### AMH measurements

AMH concentration was measured from archived serum samples of women using picoAMH ELISA kits from AnshLabs. To ensure precision and consistency, sample preparation and ELISA bioassays were conducted using an automated liquid handler (Opentrons OT-2), minimizing operator variability and improving coefficients of variation (CV). Samples from participants aged 50 years and older were analyzed neat, while those from participants younger than 50 years were diluted 7X. Each study sample was prepared and analyzed in duplicate across two identical ELISA plates, with calibration curves and quality control samples also assessed in duplicate to ensure accuracy, according to manufacturer’s instructions. If a sample’s absorbance remained below the lowest calibrator when analyzed neat, the AMH concentration was recorded as non-detectable. All samples had AMH levels below 4,700 pg/mL, a threshold associated with the diagnosis of polycystic ovary syndrome (PCOS) (39).

### Covariates

Maternal age (in years) at one month postpartum and at AMH measurement, maternal level of education at baseline (<high school, high school, and >=high school), exposure to second-hand smoke at home (yes/no), parity (1, ≥2), and breastfeeding in the first month postpartum (never breastfeed, attempted/started but did not sustain, non-exclusive, and exclusive) were included as covariates. We also included mother’s body mass index (BMI) pre-pregnancy and AMH measurement, and plate sample. These variables were used as covariates due to their association with AMH levels in previous publications (7, 12, 13, 40).

### Statistical analysis

We performed descriptive analysis for all the variables included in the analysis. Continuous variables are reported as either mean ± standard deviation (SD) or median with interquartile range (IQR), and categorical variables are expressed as percentage (%). We assessed the correlation between log_2_-transformed AMH concentration and log_2_-transformed serum stress hormone levels using Spearman’s correlation. We used scatterplot to visualize the relationship between serum stress hormones and AMH concentration and then overlaid a LOWESS (locally weighted scatterplot smoothing) curve to identify potential non-linear trends.

We calculated the absolute difference between cortisol and cortisone (F/E ratio) and determined its association with AMH concentration. A high F/E ratio indicates reduced inactivation of cortisol due to decreased 11β-HSD2 activity, resulting in higher circulating levels of cortisol relative to cortisone (41).

We used a multivariable linear regression model with natural cubic spline transformation to evaluate the adjusted association between serum stress hormone and AMH concentration at the 4- and 10-year follow-ups, adjusted for covariates. For the analysis considering both time points, we used a mixed-effects linear regression model with a random intercept per participant to assess the association between serum stress hormones and AMH levels, adjusted for covariates, considering repeated measurements between the two time points. Due to the evidence of non-linear relationship between serum stress hormones and AMH concentration, we categorized serum stress hormones in tertiles (low, moderate, and high), allowing us to simplify the interpretation and determine the category at a higher risk of accelerated ovarian aging. For participants with repeated measures (*n* = 85), we also calculated the absolute difference in AMH concentration between 4- and 10-year follow-ups and determined its association with serum stress hormones. Statistical significance was determined based on 95% confidence intervals (95% CIs) and two-sided *P*-values <0.05. All analyses were performed using R studio, version 4.3.2 (2023-10-31).

## Results

Table 1 shows the characteristics of the study population. The participants were, on average, 29.5 years old (SD: 5.2) at one month postpartum, and 34.5 (SD: 5.4) and 41.3 (SD: 4.7) at the 4- and 10-year follow-up visits. Over 24% of mothers were primiparous, 47.7% had primary level of education, 13.1% were exposed to second-hand smoke at home at baseline, and 54.2% practiced non-exclusive breastfeeding. Mother’s BMI pre-pregnancy averaged 27.3 (SD: 4.0) and varied slightly from 28.1 (SD: 5.2) to 29.5 (SD: 4.9) at the 4- and 10-year follow-up visits. The median perceived stress during the third trimester of pregnancy was 5 (IQR: 0–12), while for negative life events, it was 3 (IQR: 0–10). Log_2_-transformed serum cortisol abundance levels averaged 16.2 (SD: 0.6) and ranged from 14.7 to 17.7, while serum cortisone averaged 15.5 (SD: 0.6) and ranged from 14.1 to 16.8. Cortisol and cortisone measures are unitless because they are obtained from an untargeted assay. AMH concentration ranged from 10.0 to 2,204.9 pg/mL and averaged 593.0 (SD: 484.2) pg/mL at the 4-year follow-up visit, while at the 10-year follow-up, AMH concentration ranged from 3.8 to 3,804.3 pg/mL and averaged 360.0 (SD: 527.4) pg/mL (Table 1).

**Table 1:** Characteristics of the study population (*n* = 107 participants).

Variables	Values, *n* (%)
Maternal age (in years) at baseline	29.5 (5.17)
Maternal age (in years) after 4 years postpartum	34.5 (5.4)
Maternal age (in years) after 10 years postpartum	41.3 (4.7)
Parity	
1	26 (24.3)
≥2	81 (75.7)
Maternal education	
<High school	51 (47.7)
High school	35 (32.7)
>=High school	21 (19.6)
Maternal exposure to smoke inside the home	
Yes	14 (13.1)
No	93 (86.9)
Breastfeeding in the first month postpartum	
Never breastfeed	2 (1.9)
Attempted/started but did not sustain	6 (5.6)
Non-exclusive	58 (54.2)
Exclusive	41 (38.3)
Negative life events during pregnancy	3 (range: 0–10)
Perceived stress during pregnancy	5 (range: 0–12)
Mother’s BMI pre-pregnancy	27.3 (4.0)
Mother’s BMI after 4 years postpartum	28.1 (5.2)
Mother’s BMI after 10 years postpartum	29.5 (4.9)
Serum cortisol	16.2 (SD: 0.6)
	16.2 (IQR:14.7–17.7)
Serum cortisone	15.5 (SD: 0.6)
	15.5 (IQR:14.1–16.8)
Ratio of cortisol to cortisone	0.70 (SD: 0.74)
	0.65 (IQR:−0.8;2.4)
AMH concentration (pg/mL) at
4 years postpartum	
Mean (SD)	593.0 (484.2)
Interquartile range	10.0–2,204.9
AMH concentration (pg/mL) at
10 years postpartum
Mean (SD)	366.0 (527.4)
Interquartile range	3.8–3,804.3

AMH, anti-Müllerian hormone; BMI, body mass index; IQR, interquartile range; SD, standard deviation.

There was a negative correlation between the log_2_-transformed AMH concentration and mother’s age at the time of AMH data collection (rho = −0.46, *P* < 0.001, and rho = −0.38, *P* < 0.001, at the 4- and 10-years follow-ups, respectively; Supplementary Fig. 1A and 1B (see section on Supplementary materials given at the end of the article)). At the 4-year follow-up, serum cortisone was positively correlated with AMH concentration (rho = 0.30, *P* = 0.002), while F/E ratio showed a negative correlation with AMH concentration (rho = −0.26, *P* = 0.007; Supplementary Fig. 2A, 2B, 2C). At the 10-year follow-up, similar results were observed between serum cortisone and AMH concentration (*r* = 0.33, *P* = 0.002) and between F/E ratio and AMH concentration (*r* = −0.27, *P* = 0.012), but the correlation between serum cortisol and AMH concentration was not statistically significant (Supplementary Fig. 3A and 3B). Exploratory analysis using LOWESS smoothing scatterplot demonstrated a deviation from linearity in the relationship between serum stress hormones and AMH concentration (Supplementary Figs 2A, 2B, 2C, and 3A, 3B, 3C).

### Association between serum stress hormones and log_2_-transformed AMH concentration

**Figure 1:**
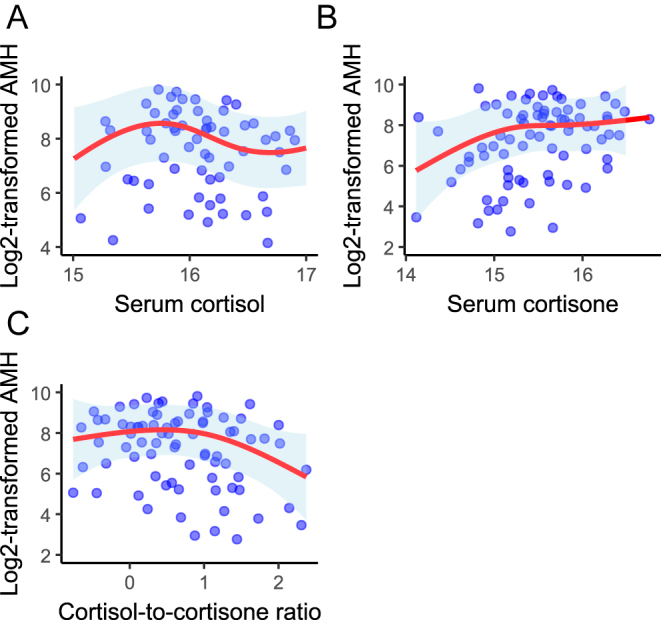
Association between serum stress markers cortisol, cortisone, and their ratio and the log_2_-transformed AMH concentration after 10 years postpartum from the multivariate linear mixed models with natural cubic spline transformation (*n* = 85). Notes: AMH, anti-Müllerian hormone. The analyses were adjusted for maternal age at 1 month and at AMH measurement, maternal level of education, exposure to second-hand smoke at home, BMI pre-pregnancy, parity, plate sample, and breastfeeding status at 1 month postpartum.

After covariate adjustment, we observed a non-significant, but inverse association between one month postpartum serum cortisol (*β* = −0.46, 95% CI: −0.97; 0.06, *P* = 0.08) and AMH concentration at the 4-year follow-up and between the F/E ratio (*β* = −0.31, 95% CI: −0.72; 0.10, *P* = 0.083) and AMH concentration during the same period (Supplementary Fig. 4A, 4B, 4C). There was no statistically significant association between one month postpartum cortisol (*β* = −0.37, 95% CI: −1.08; 0.33) and AMH levels at the 10-year follow-up (Fig. 1A). However, each unit increase in serum cortisone at one month postpartum was associated with a 114% increase in log_2_-transformed AMH concentration (*β* = 0.76, 95% CI: 0.01; 1.52) 10 years later (Fig. 1B). In contrast, each unit increase in the F/E ratio was associated with a 45% lower AMH concentration 10 years later (*β* = −0.60, 95% CI: −1.13; −0.07) (Fig. 1C). Analysis with both time points also showed that each unit increase in the F/E ratio was associated with a 32% decrease in AMH concentration (*β* = −0.38, 95% CI: −0.75; −0.01; Supplementary Table 1). We performed an interaction test between serum stress hormones and maternal age at AMH measurement, which showed no statistically significant interaction (*P*-value >0.05). The analyses of serum stress hormones by tertiles showed a lower AMH concentration among women in the highest tertile of F/E ratio (*β* = −0.75, 95% CI: −1.46; −0.04; Table 2) at the 4-year follow-up. At 10 years, there was an increase in AMH concentration with serum cortisone in the second (*β* = 1.18, 95% CI: 0.22; 2.15) and third (*β* = 1.15, 95% CI: 0.14; 2.16) tertiles, but a decline in the highest tertile of F/E ratio (*β* = −1.23, 95% CI: −2.18; −0.28) (Table 3). A statistically significant decrease was observed in AMH concentration in the highest tertile of F/E ratio in the analysis with both time points (*β* = −0.91, 95% CI: −1.54; −0.28; Supplementary Table 1). Analysis of the absolute change in AMH levels between the two points (*n* = 85) showed a decrease in AMH levels per unit increase in serum cortisol, although the associations were not statistically significant (Supplementary Table 2). There was a positive association between serum cortisone levels and AMH levels in the model without the inclusion of maternal age (*β* = 0.89, 95% CI: 0.16; 1.62). However, after including maternal age, the model was no longer statistically significant. Similarly, in the model without the inclusion of maternal age, a negative association was observed between the F/E ratio and the absolute change in AMH levels (*β* = −0.55, 95% CI: −1.07; −0.04), which disappeared after the inclusion of maternal age in the model (Supplementary Table 2).

**Table 2:** Average changes (*β* (95% CIs)) in log_2_-transformed AMH concentration according to tertiles of serum stress markers cortisol, cortisone, and their ratio at 4-year follow-up from the multivariable linear regression models.

Stress hormone compounds	Tertile 1	Tertile 2	Tertile 3
Cortisol (T1 = 29, T2 = 29, T3 = 27 participants)	Ref	−0.66 (−1.37; 0.05)	−0.61 (−1.34; 0.13)
Cortisone (T1 = 28, T2 = 26, T3 = 31 participants)	Ref	−0.35 (−1.01; 0.31)	0.06 (−0.67; 0.79)
F/E ratio (T1 = 28, T2 = 32, T3 = 25 participants)	Ref	0.14 (−0.54; 0.81)	**−0.75 (−1.46; −0.04)**

Analyses adjusted for maternal age at 1 month and at AMH measurement, maternal level of education, exposure to second-hand smoke at home, BMI pre-pregnancy, parity, plate sample, and breastfeeding status at 1 month postpartum. T1, tertile 1; T2, tertile 2, and T, tertile 3. Significant findings are in bold.

**Table 3:** Average changes (*β*  (95% CIs)) in log_2_-transformed AMH concentration according to tertiles of serum stress markers cortisol, cortisone, and their ratio at 10-year follow-up from the multivariable linear regression models.

Stress hormone compounds	Tertile 1	Tertile 2	Tertile 3
Cortisol (T1 = 29, T2 = 29, T3 = 27 participants)	Ref	−0.26 (−1.24; 0.71)	−0.92 (−1.89; 0.06)
Cortisone (T1 = 28, T2 = 26, T3 = 31 participants)	Ref	**1.18 (0.22; 2.15)**	**1.15 (0.14; 2.16)**
F/E ratio (T1 = 28, T2 = 32, T3 = 25 participants)	Ref	0.34 (−0.53; 1.22)	**−1.23 (−2.18; −0.28)**

Analyses adjusted for maternal age at 1 month and at AMH measurement, maternal level of education, exposure to second-hand smoke at home, BMI pre-pregnancy, parity, plate sample, and breastfeeding status at 1 month postpartum. T1, tertile 1; T2, tertile 2, and T, tertile 3. Significant findings are in bold.

## Discussion

This study explored the association between serum stress-related hormones at one month postpartum and ovarian aging, as measured by AMH concentrations, up to 10 years later. We observed a negative association between the F/E ratio and AMH levels at 4- and 10-year follow-ups, as well as in the repeated measure analysis including both time points, while a positive association was observed between serum cortisone at one month postpartum and AMH levels 10 years later. Our findings align with the notion that dysregulated stress hormone dynamics during the early postpartum period can have long-term implications for later reproductive endocrine health (42). A higher F/E ratio indicates an imbalance in the activity of 11β-HSD enzymes, which regulate the interconversion between biologically active cortisol and its inactive form, cortisone (43, 44). This imbalance could reflect disrupted HPA axis function, which might accelerate functional ovarian aging, resulting in a decreased AMH concentration.

The analysis of serum stress hormones by tertiles showed that only women in the highest tertile of F/E ratio had significantly lower AMH levels compared to women in the lowest tertile. This suggests a non-linear relationship between stress-related hormones and ovarian aging, as well as the presence of a threshold effect where the detrimental impact of stress-related hormonal activity on ovarian reserve becomes more evident. Such a non-linear relationship may reflect a physiological tolerance for moderate hormonal stress exposure, while excessive activation potentially disrupts reproductive function and accelerates ovarian aging.

Furthermore, we observed a significant association between serum stress markers (serum cortisol and F/E ratio) and the absolute difference in AMH concentration between the two time points, excluding maternal age from the model. This suggests that the observed effect is partially explained by maternal age, which acted as a confounding factor. The interaction test between serum stress hormones and AMH levels was not statistically significant, either in the subgroup assessed at 4 years or in the subgroup assessed at 10 years of follow-up, or when repeated measures are considered. This indicates that maternal age did not modify the association between serum stress hormones and AMH levels.

In summary, the negative association between the F/E ratio at one month postpartum and AMH levels ten years later combined with the positive association between serum cortisone at one month postpartum and AMH ten years later suggests a protective effect of cortisone, and potentially rapid conversion of cortisol to cortisone, on future functional ovarian reserve. This emphasizes the importance of hormonal balance in maintaining reproductive health and preventing premature AMH decline. Since elevated cortisol is an indicator of chronic stress (45) and likely reflects inefficient stress hormone clearance (46), our findings further support the long-term reproductive benefits of managing stress in the months following childbirth (47, 48).

Our study has strengths and limitations. We explored the prospective association between serum cortisol, cortisone, and their ratio at one month postpartum and AMH concentration up to 10 years later, providing novel insight into postpartum period as a critical window for the effect of stress exposure on women’s long-term reproductive health. Given that the postpartum period is considered one of the most stressful periods in a woman’s life with profound hormonal fluctuations, disrupted sleep, intensive caregiving demands, among others (49), these stressors when combined with inadequate social support and other negative life events may disrupt normal HPA axis regulation (50) and have lasting effects on ovarian aging. The use of serum stress-related hormones instead of self-reported psychosocial stress in this study offers a more objective and biological measure of stress response, capturing physiological dysregulation of the HPA axis that may not be evident through subjective stress reports. However, unitless measures of cortisol and cortisone from untargeted metabolomics data limit comparability across studies.

We controlled for several covariates, including maternal age, maternal level of education, exposure to second-hand smoking inside the home, parity and BMI pre-pregnancy, and at AMH measurement, breastfeeding status at one month postpartum, exposure to negative life events, and perceived stress during pregnancy, allowing us to minimize potential confounding effects and ensuring that the observed association is likely to reflect the true association between the stress-related hormones and AMH levels. However, the reliance on serum cortisol and cortisone measured at a single point in time may not fully capture the chronic or cumulative stress exposure across the reproductive lifespan. The relatively small sample size used in this study may also have limited the statistical power to detect a greater effect between the stress hormones and AMH levels.

## Conclusion and recommendations

Our findings suggest that an elevated cortisol-to-cortisone ratio negatively impacts AMH levels, indicating that higher cortisol may be detrimental to ovarian aging. These findings underscore the long-term effects of chronic stress on ovarian aging. Larger prospective analyses are needed to strengthen this association.

## Supplementary materials



## Declaration of interest

The authors declare that there is no conflict of interest that could be perceived as prejudicing the impartiality of the work reported.

## Funding

This study was funded by the National Institutes of Health/National Institute of Environmental Health Sciences (R01 ES036725, R01 ES014930, R01 ES013744, P30 ES023515, R01 ES031117, R01ES036725, and UL1 TR004419) and the National Institute of Public Health/Ministry of Health of Mexico.

## Author contribution statement

LP conceived the study. MMTR, GD, BPP, MY, and KS curated and verified the data. LP, ROW, MMTR, and EC acquired funding. GJ and LP developed the methodology. GJ performed formal analysis. LP supervised the study. GJ and LP wrote the original draft of the manuscript. All authors wrote, reviewed, and edited the manuscript. All authors approved the final version of the manuscript.

## Data availability

The data supporting the conclusions of this study can be obtained from the corresponding author upon reasonable request.

## Ethical approval

The study approval was obtained from the institutional review boards at the Harvard School of Public Health and Icahn School of Medicine at Mount Sinai; the Research, Ethics and Biosafety Committees at the Mexico’s National Institute of Public Health; and the National Institute of Perinatology.
