# Supplementary material for: Cortisol-to-cortisone ratio postpartum is associated with anti-Müllerian hormone a decade later: evidence from a prospective study
Source: Endocr Connect. 2026 Mar 25;15(3):e250871. doi: 10.1530/EC-25-0871 (PMC13034527; doi:10.1530/EC-25-0871)
Supplement: Supplementary file 1 [file supplementary_materials.pdf]

Cortisol-to-cortisone ratio postpartum is associated with anti-Müllerian hormone a decade years later: Evidence from a prospective study

Supplementary materials

Supplementary Figure 1: Trends in log-2 transformed AMH according to mothers ‘age after 4 and 10 years postpartum.

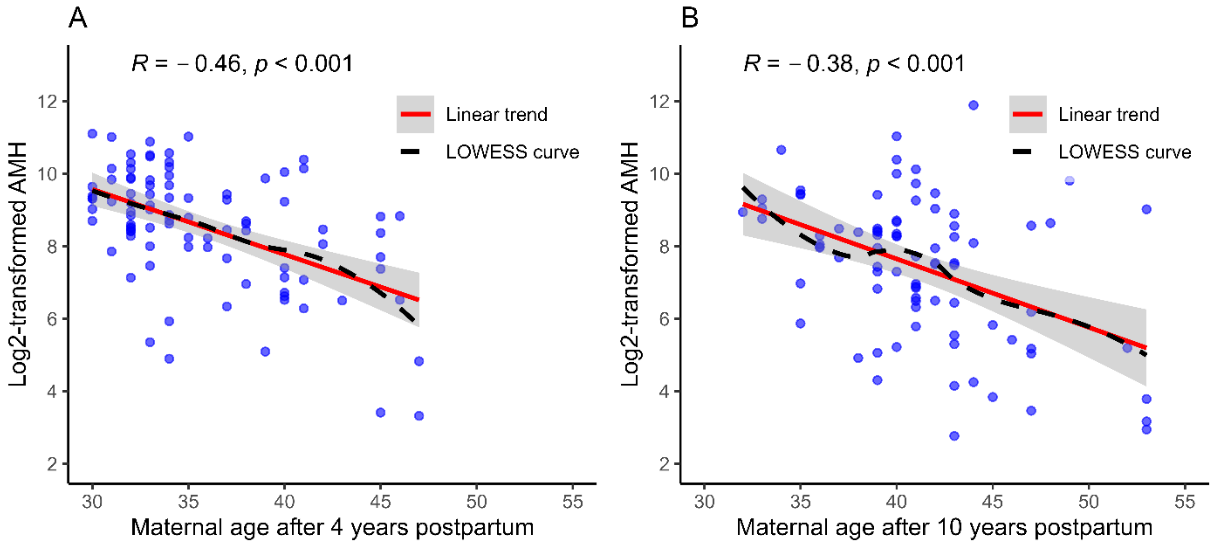

Footnotes: AMH: Anti-Müllerian Hormone

Supplemental Figure 2: LOWESS-smoothed scatter plot showing the relationship between serum stress cortisol, cortisone, their ratio and the log-2 transformed AMH after 4 years postpartum (n=107).

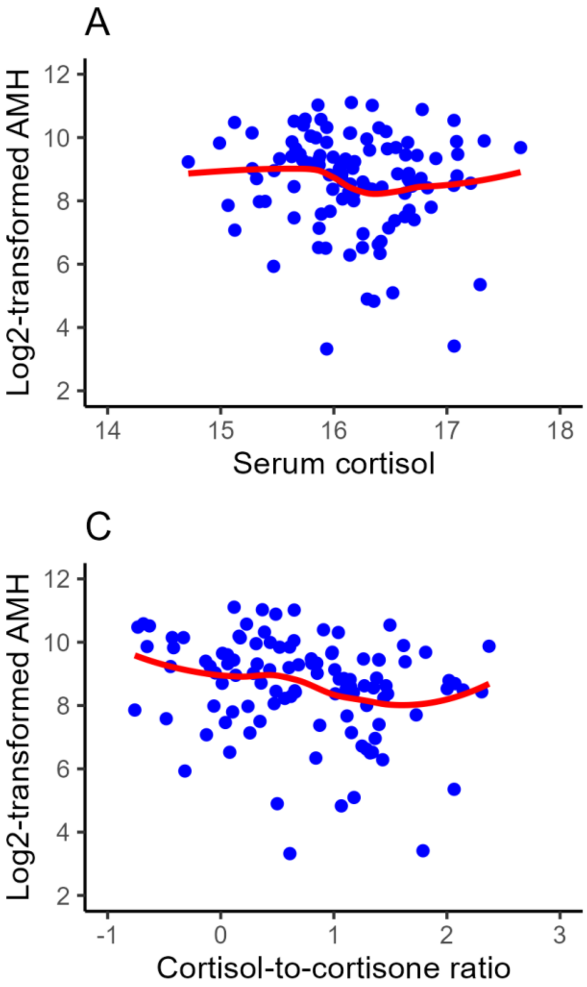

Footnotes: AMH: Anti-Müllerian Hormone

Supplemental Figure 3: LOWESS-smoothed scatter plot showing the relationship between serum stress cortisol, cortisone, their ratio and the log-2 transformed AMH after 10 years postpartum (n=85).

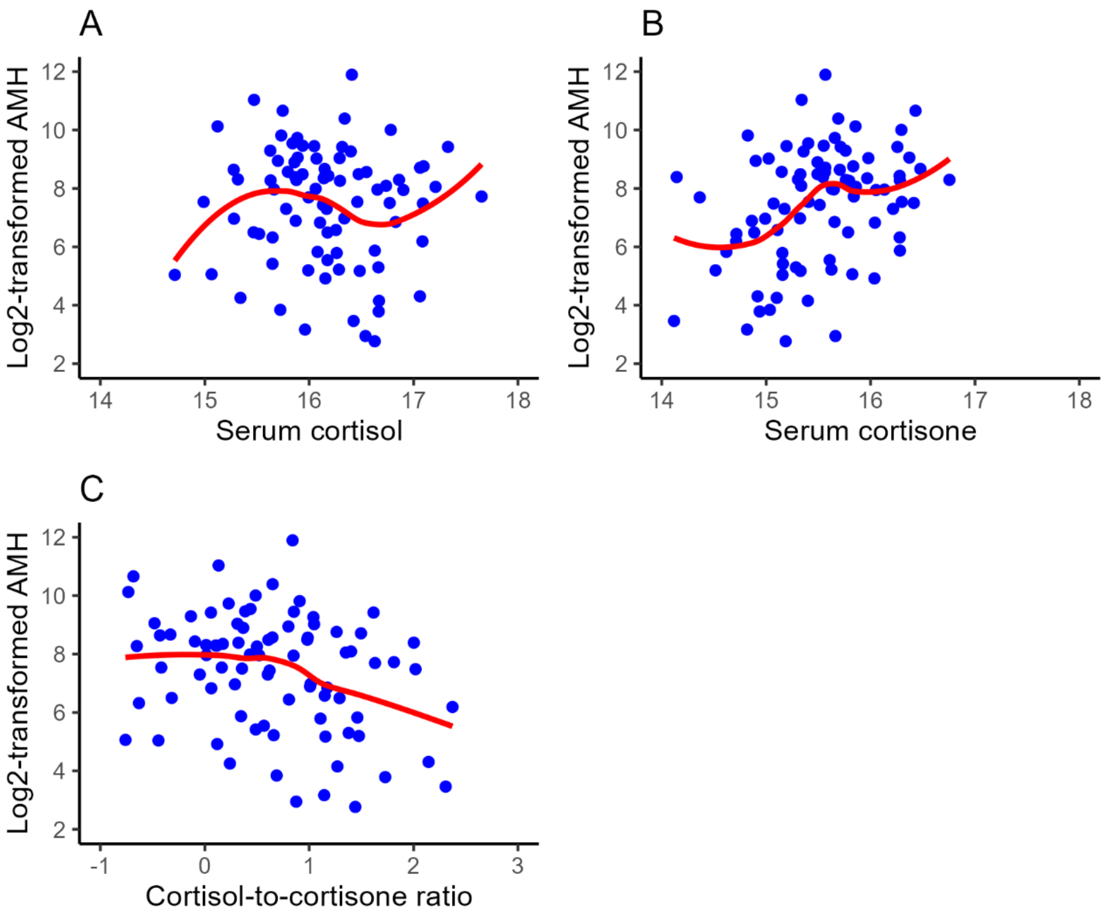

Footnotes: AMH: Anti-Müllerian Hormone

Supplementary Figure 4: Association between serum stress cortisol, cortisone, their ratio and the log-2 transformed AMH concentration after 4years postpartum from the multivariate linear models with natural cubic spline transformation (n=107).

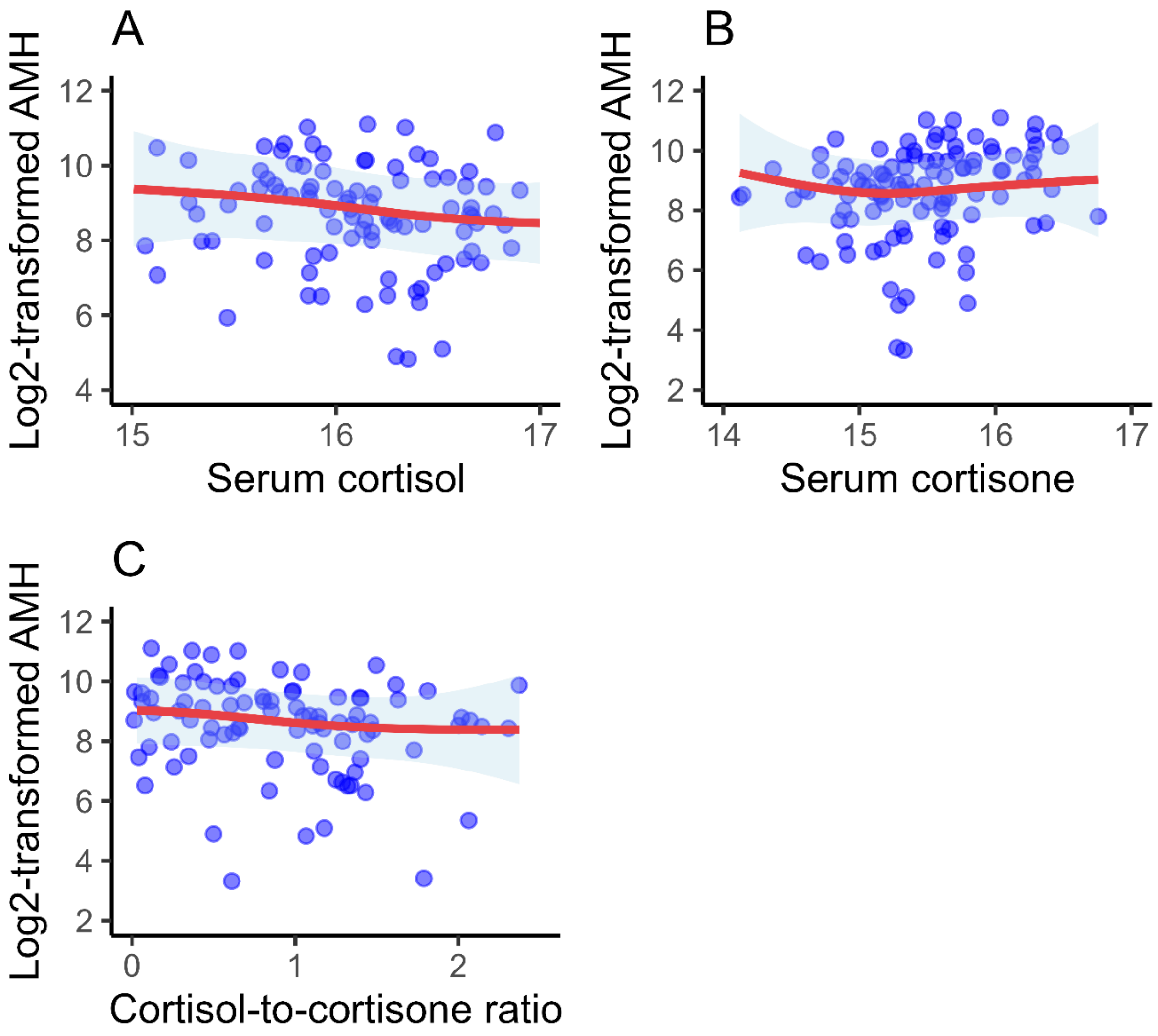

Footnotes: AMH: Anti-Müllerian Hormone. The analyses were adjusted for maternal age at 1 month and at AMH measurement, maternal level of education, exposure to second-hand smoke at home, BMI pre-pregnancy, parity, plate sample, and breastfeeding status at 1-month postpartum.

Supplementary Table 1: Association between serum stress cortisol, cortisone, their ratio and the log-2 transformed AMH concentration in the total population (n=107) from the multivariate linear mixed models.

Stress hormones compounds	Log 2 AMH levels (pg/mL)			
	All (n=192)<br>β (95%CI)	Tertile 1<br>β (95%CI)	Tertile 2<br>β (95%CI)	Tertile 3<br>β (95%CI)
Cortisol	-0.39 (-0.86; 0.07)	ref	-0.34 (-0.99; 0.30)	-0.61 (-1.28; 0.06)
Cortisone	0.38 (-0.13; 0.90)	ref	0.30 (-0.32; 0.92)	0.56 (-0.10; 1.23)
F/E ratio	**-0.38 (-0.75; -0.01)**	ref	0. 23 (-0.36; 0.81)	**-0.91 (-1.54; -0.28)**

Footnotes: Analyses adjusted for maternal age at 1 month and at AMH measurement, maternal level of education, exposure to second-hand smoke at home, BMI pre-pregnancy, parity, plate sample, and breastfeeding status at 1-month postpartum. T1= Tertile 1, T2= Tertile 2, and T= Tertile 3. Significant findings are in bold. Cortisol (T1=63, T2=67, T3=62) , Cortisone (T1=64, T2=64, T3=64), F/E ratio (T1=63, T2=65, T3=64).

Supplementary Table 2: Association between serum stress cortisol, cortisone, their ratio, and the absolute difference in log-2 transformed AMH concentration after 4 and 10-year follow-up in the total population (n=85) from the multivariate linear models.

Stress hormones compounds	Log 2 AMH levels (pg/mL)		
	Model 1: Unadjusted	Model 2: Adjusted (without maternal age) *	Model 3: Fully adjusted**
	β (95% CI)	β (95% CI)	β (95% CI)
Cortisol	-0.12 (-0.86; 0.62)	-0.20 (-0.91; 0.51)	-0.14 (-0.80; 0.52)
Cortisone	**1.09 (0.38; 1.81)**	**0.89 (0.16; 1.62)**	0.48 (-0.25; 1.23)
F/E ratio	**-0.68 (-1.22; -0.14)**	**-0.55 (-1.07; -0.04)**	-0.32 (-0.83; 0.19)

Footnotes: *Analyses adjusted for maternal level of education, exposure to second-hand smoke at home, mother's BMI, parity, plate sample, and breastfeeding status at 1-month postpartum. **Analyses adjusted for maternal age at 4 postpartum, maternal level of education, exposure to second-hand smoke at home, mother's BMI, parity, plate sample, and breastfeeding status at 1-month.
